# A Low-Cost All-Suture Anchor: A Novel Technique With Mechanical Test Validation in Osteoporotic Bone Models

**DOI:** 10.7759/cureus.83077

**Published:** 2025-04-27

**Authors:** Dominique B Spence, Natalia McIver, Christina Salas, Naga Suresh Cheppalli

**Affiliations:** 1 Department of Orthopaedics and Rehabilitation, University of New Mexico School of Medicine, Albuquerque, USA; 2 Department of Orthopaedics and Rehabilitation, Veterans Affairs (VA) Hospital, Albuquerque, USA

**Keywords:** all-suture anchor, biomechanics, osteoporotic, rotator cuff tears, tendon biomechanics

## Abstract

Achieving higher-quality health care while reducing costs is an ideal goal for health care systems throughout the globe. Commercially available suture anchor costs can be prohibitive in some under-resourced settings. This disparity highlights the need for innovative solutions that maintain high clinical efficacy while being economically accessible.

In the present study, we developed a novel all-suture anchor (ASA) design using the same general placement procedure, but utilizing materials that are available in standard operating rooms. A 2.0 braided polyblend suture was woven through a 2.5 cm-long piece of 5 mm-wide Mersilene tape, leaving two loose ends of suture on either end of the tape. We deployed this ASA into a 10-pound/cubic foot (PCF) Sawbones^®^ block (Pacific Research Laboratories, Inc., Vashon, WA, USA), representing osteoporotic bone, using a custom-made, autoclavable deployment tool. Anchors were tested at 90-degree and 45-degree angles in a custom fixture under uniaxial biomechanical tension. Test samples were first preloaded to 1 N, preconditioned via cyclic loading, and then loaded to failure to assess the maximum pullout force required for failure of each ASA. A continuous outcome non-inferiority statistical analysis was used to compare test outcomes to historical data published on commercially available ASAs, tested using similar-density material and the same insertion and loading angles. We were able to achieve our aim of creating a low-cost ASA from existing operating room materials. These anchors were not statistically different from the maximum pullout force values of commercial ASA products previously tested. This alternate soft anchor may be a viable option for those in need of a low-cost solution, but further material options, deployment techniques, and loading angles must be explored before cadaveric testing is done.

## Introduction

Suture anchors are fixtures implanted into bone to reattach soft tissue after injury and have undergone evolutionary changes over time. Various materials, including metal and polymeric anchors, have been utilized as suture anchors, leading to the development of biodegradable options [1]. Recently, all-suture anchors (ASAs) have been developed to address some of the shortcomings of previous designs, such as the preservation of bone stock, the ability to use more anchors in smaller areas, reduced risk of implant-related complications, lower profile, versatility, abrasion of suture materials, corrosion, and metal allergies. Commercially available ASAs feature different designs and patterns, but they commonly function by having the suture bunch up against the cortex, acting as a soft cortical suspensory device.

The cost of procedures involving suture anchors can vary significantly depending on the institution and its ability to contractually negotiate with major companies, the number of anchors used, and the deployment tools required - often escalating to several thousand dollars [2-4]. To address these variables, we have designed a low-cost ASA using readily available materials, such as Mersilene tape and high-strength suture, employing a simple and reproducible technique that any surgical technician can perform. This approach enables the production of multiple anchors (four to five) for the surgeon's use within a short time frame (approximately two to five minutes). Our objective is to create an affordable ASA from standard operating room materials that meets or exceeds the pullout forces of comparable, commercially available ASA products.

## Technical report

Materials and methods

Suture Anchor Design

**Figure 1:**
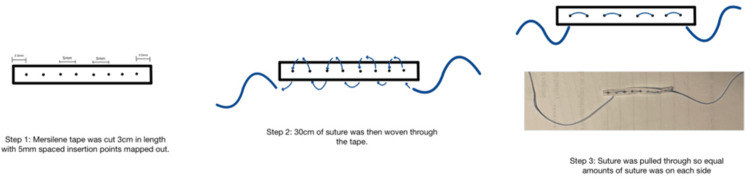
Novel all-suture anchor design with step by step guide for replication Image credit: Dominique B. Spence

The suture anchor design utilizes readily available materials in standard operating rooms. Our tested design used a 5 mm broad white woven Ethicon Mersilene Tape (Ethicon, Inc., Raritan, NJ, USA) - polyester fiber ligature as the anchor. To test the hypothesis that virtually any materials could be used to create this anchor system, we created and tested two suture anchor systems for this study: for the first, we used #2 FiberWire^®^ AR-7200 Suture (Arthrex, Inc., Naples, FL, USA), constructed of long-chain ultra-high molecular weight polyethylene (UHMWPE) core with a braided jacket of polyester and UHMWPE; and the second, with flat #2 BroadBand™ Suture Tape (Zimmer Biomet Holdings, Inc., Warsaw, IN, USA), constructed from a proprietary braid and coreless UHMWPE weave of Max Braid and TRU-Link sutures (Zimmer Biomet Holdings, Inc.) [5,6]. To construct the anchor, the Mersilene tape was cut into 3 cm strips, and the suture was cut to 30 cm in length. The suture was woven through the Mersilene tape with 5 mm spacing between each insertion, and six insertion points total. To standardize the tested anchors, each one was made by the same personnel, who precisely measured the placement of all insertion points (Figure 1).

**Figure 2:**
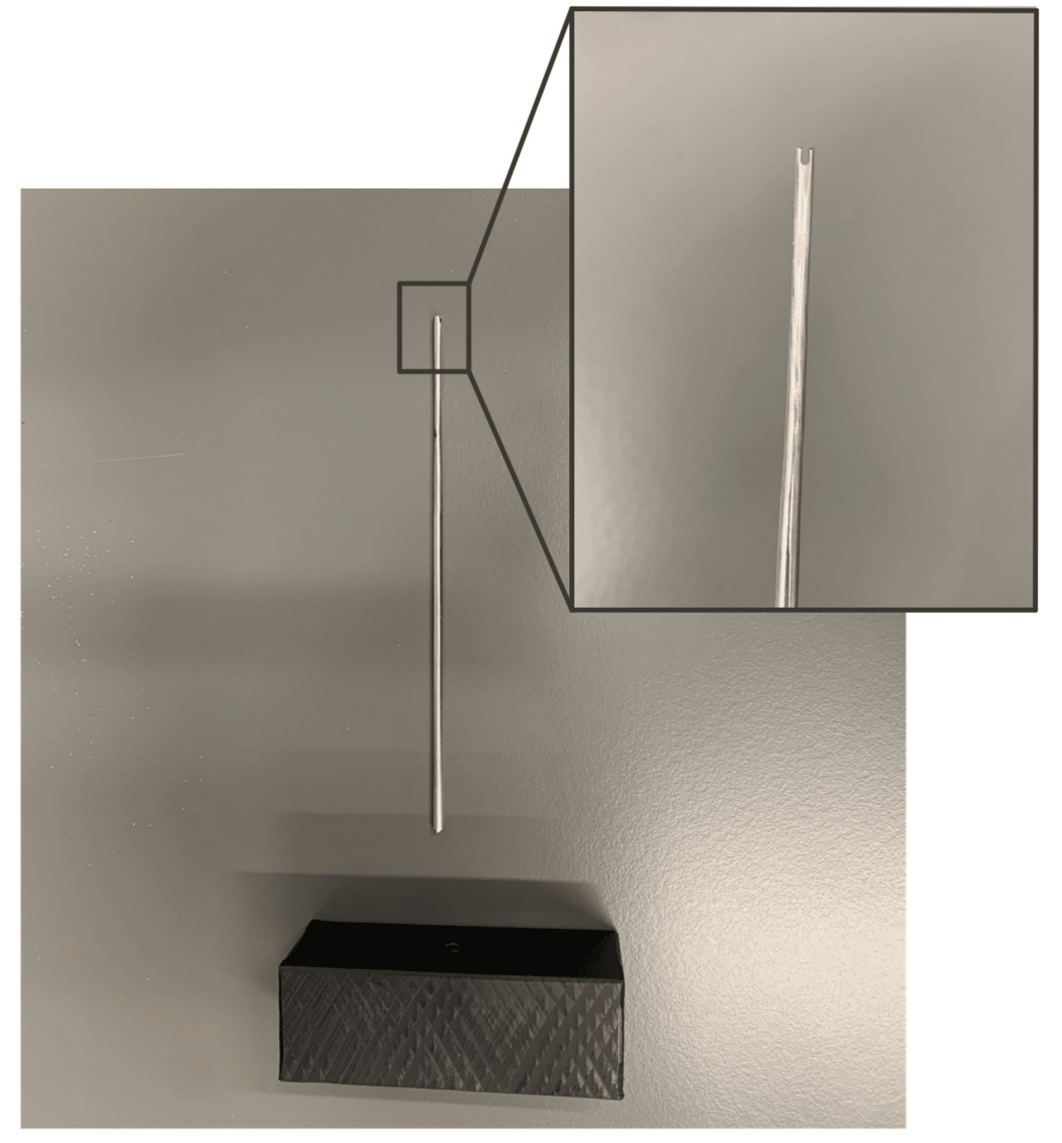
Novel deployment tool was created using a modified Kirschner wire and an ABS block Image credit: Dominique B. Spence ABS, Acrylonitrile Butadiene Styrene

A deployment tool was created using a 2.5 mm Kirschner wire that was notched to form a tuning fork end. A 2.5 mm hole was drilled to a 10 mm depth in a 25 x 75 x 50 mm acrylonitrile butadiene styrene (ABS) block to create a handle. The Kirschner wire was then placed into the ABS box and served as a deployment tool that can easily be re-sterilized for multiple uses (Figure 2).

Experimental Test Design

**Figure 3:**
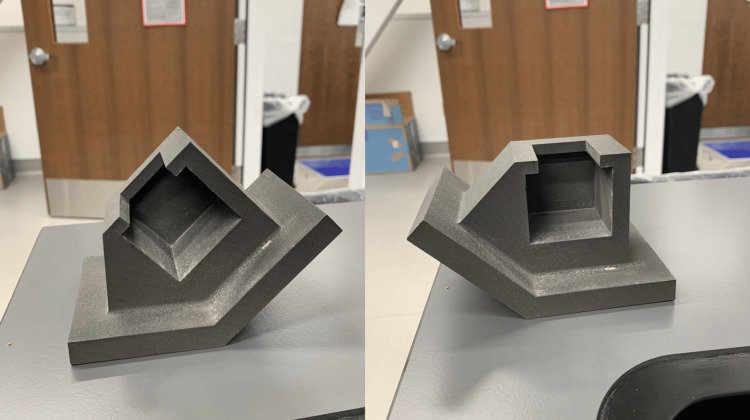
Selective laser sintered (3D printed) fixture, capable of being tested at 45-degree (left) and 90-degree (right) angles Image credit: Dominique B. Spence and Natalia McIver

To test the pullout force of the created suture anchors, a 10-pound/cubic foot (PCF) Sawbones^®^ solid foam block (Pacific Research Laboratories, Inc., Vashon, WA, USA), with a 1 mm thick laminated epoxy layer, was used to simulate osteoporotic bone with a thin cortical layer [3]. The Sawbones^®^ block was cut into 42 x 45 x 40 mm pieces to fit into the custom test fixture. Four 2.5 mm pilot holes were drilled into the block, with 2 cm separation between each hole. A custom Nylon 12 selective laser sintered (3D printed) fixture was created to hold the Sawbones^®^ block and allow for testing at 90-degree and 45-degree loads (Figure 3). This fixture was mounted to a plate on the base of an Instron 5940 (Instron Corporation, Norwood, MA, USA) load frame, equipped with a 1 kN axial load cell. To evaluate the load capability of the suture, we first inserted the suture anchor into one of the pilot holes of the Sawbones^®^ block using the Kirschner wire-adapted deployment tool. The suture was placed flat over the hole and pushed in perpendicularly using the tool. Once the Mersilene tape was completely embedded, the suture strings were pulled back and forth to collapse and bunch up the tape, ensuring the suture anchor was fully deployed.

**Figure 4:**
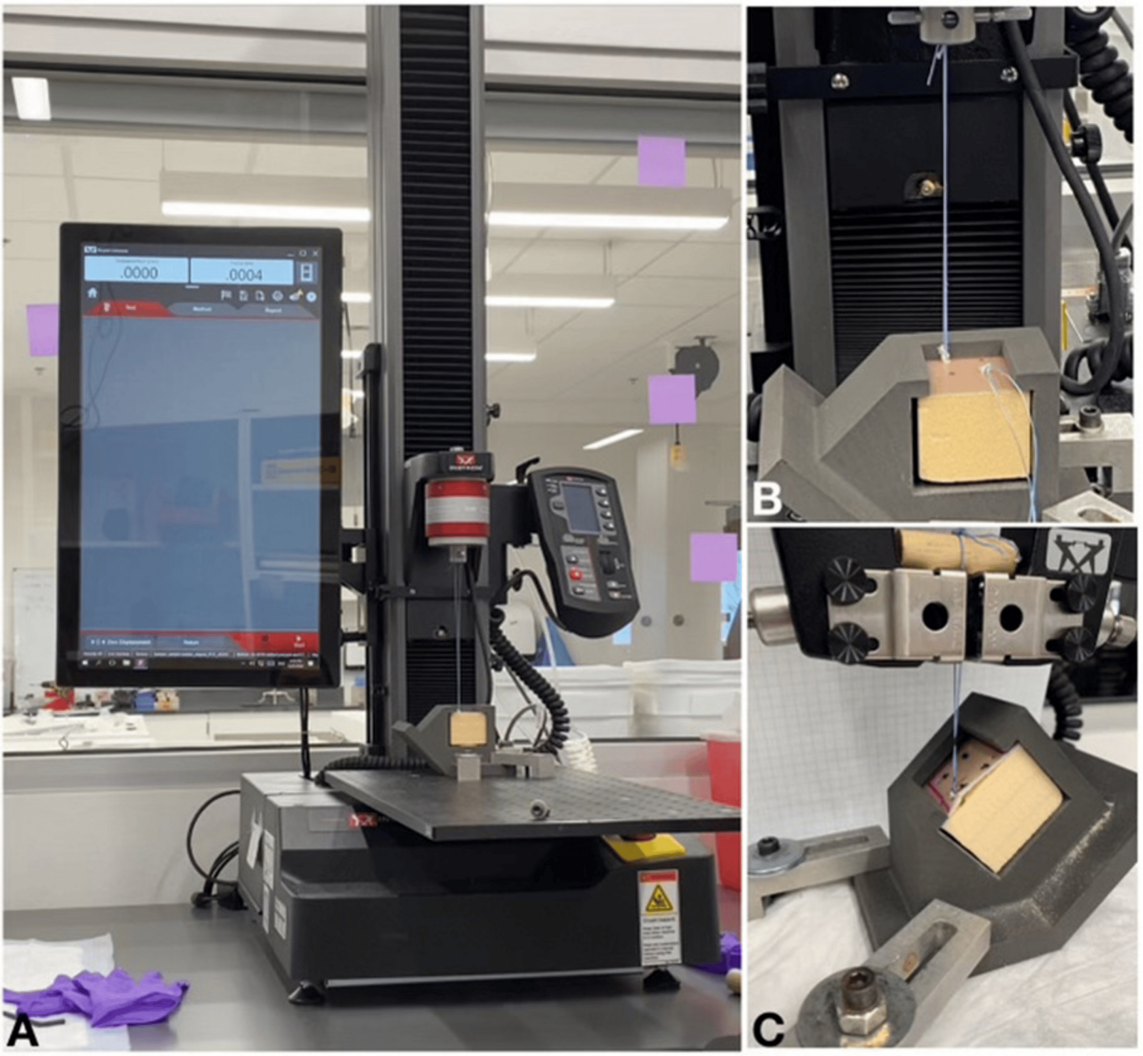
Experimental testing setup A) Instron 5940 load frame with loaded novel all-suture anchor in fixture; B) Deployed novel all-suture anchor at a 90-degree angle; C) Deployed novel all-suture anchor at a 45-degree angle, with dowel adaptation to overcome suture slippage. Image credit: Dominique B. Spence and Natalia McIver

The suture ends were tied off with eight surgical knots to establish stability and create a loop to be used for axial loading. The block was then placed in the fixture at the chosen angle and attached to the Instron machine using the surgical knot (Figure 4). The suture was first preloaded to 1 N and then cyclically loaded from 10 N to 50 N at 0.5 Hz for 200 cycles [2]. It was then ramped at 10 mm/s in tension until failure. The failure load is determined by a loss of force of 50% of the maximum load. The failure load was then recorded and compared to published outcomes of commercially available products that were tested under similar conditions. We also captured data on the mode of failure and survival rate during cyclic loading. We tested both ASA constructs generated from the FiberWire^®^ and BroadBand™ Tape, respectively.

A continuous outcome non-inferiority statistical analysis was used to compare test outcomes to historical data of commercially available soft anchor systems. The non-inferiority margin (-Δ) was set at -25 N. This type of analysis is often used to conclude whether a new treatment is not unacceptably different, quantitatively, from an existing treatment and may, in fact, have qualitative advantages over the existing options. In the present study, we compared outcomes to the studies done by Douglass et al. and Oh et al. [2,3], but were careful to only use results from samples tested at 10 PCF and 20 PCF, representative of osteoporotic or normal bone density. We were also careful to only compare our findings to outcomes tested at 90-degree and 45-degree insertion and loading angles, as appropriate for each comparison.

Results

*Suture Anchors Made With FiberWire^®^* *Suture*

**Figure 5:**
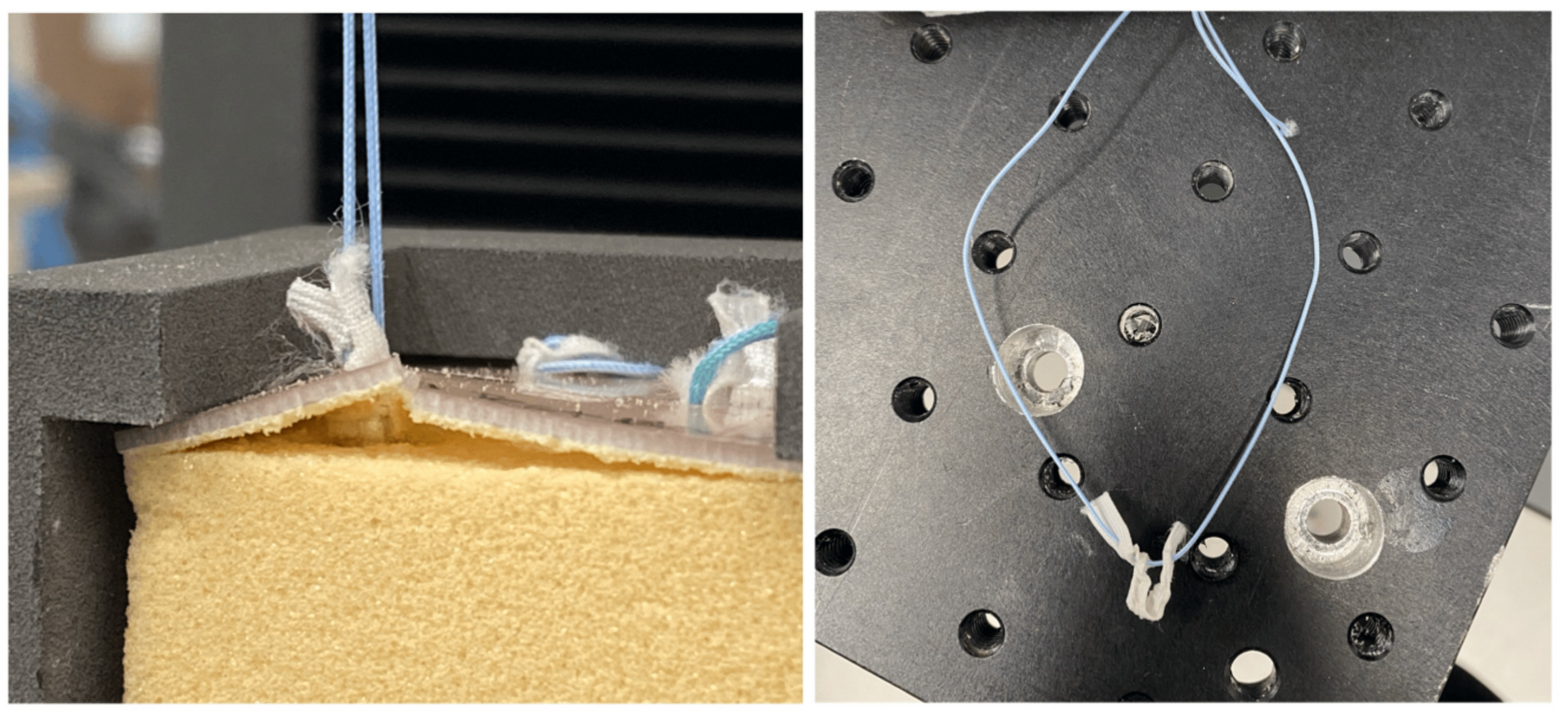
Failure types Left: Failure by pullout, where the anchor has breached the cortical top layer; Right: Failure by suture breakage through the Mersilene tape construct. Image credit: Dominique B. Spence and Natalia McIver

A total of 20 novel ASAs were tested, made with the FiberWire^®^ Suture: 10 at a 90-degree loading angle and 10 at a 45-degree loading angle. In the 90-degree test, all samples survived cyclic loading to 50 N. During ramped displacement, nine of the anchors failed by pullout, while one failed due to suture breakage. The maximum failure force of the 90-degree tests was 193.4 ± 47.2 N. In the 45-degree loads, all 10 samples survived the cyclic loading phase. The maximum failure force in this sample was 233.6 ± 35.4 N. Four failed via anchor pullout, while six failed via suture breakage or grip failure (Figure 5). When evaluating only those failed by pullout at 45 degrees, the maximum force was 242.8 ± 50.2 N. Unfortunately, the analysis for the 45-degree loading scenario was underpowered because the historical study only tested n = 5 samples [3]. A 95% confidence interval and p-values for each comparison of the respective commercial product from historically published studies, in comparison to our results, are found in Table 1.

**Table 1:** Continuous outcome non-inferiority statistical analysis results A comparison of testing our all-suture anchor (made with BroadBand™ suture tape) to commercially available products. Maximum force values are taken from published findings reported in Oh et al. and Douglass et al. [2,3]. An unpaired, two-tailed t-test was used to calculate p-values, comparing the means/SD from published data to the maximum force values obtained from our experimental testing for each matching insertion angle (175.82 ± 70.45 N at 90 degrees and 261.96 ± 49.44 N at 45 degrees, respectively). Significance was noted if the p-value was less than 0.05. *statistically significant PCF, Pounds per Cubic Foot; DF, Degrees of Freedom; CI, Confidence Interval

Block and Anchor Insertion Relative to Load Direction	Block Density (PCF)	Commercial Product	Maximum Force (N)	95% CI	t-statistic (DF)	p-value
90°	20	Iconix 25	196.0 ± 15.9	-30.5 to 35.7	0.165 (18)	0.87
		Juggerknot 2.9	193.8 ± 20.2	-33.7 to 34.5	0.025 (18)	0.98
		Iconix 3	180.0 ± 16.0	-46.5 to 19.7	-0.850 (18)	0.41
		Y-Knot Flex 1.7	176.1 ± 18.0	-50.9 to 16.26	-1.083 (18)	0.29
	10	OmegaKnot	181.9 ± 13.7	-58.8 to 35.8	-0.525 (13)	0.61
45°	10	OmegaKnot	167.8 ± 20.4	-103.1 to -28.5	-3.807 (13)	0.002*

Suture Anchors Made With BroadBand™ Suture Tape

Secondary testing was also done with another set of 16 novel ASAs made with BroadBand™ Suture Tape, with 10 tested at a 90-degree angle and 6 at a 45-degree angle. In the 90-degree test, only 90% of the samples (n = 9) survived cyclic loading to 50 N. Overall, eight of the anchors failed by pullout, while one failed due to suture breakage. The maximum failure force of the 90-degree tests was 175.82 ± 70.45 N. In the 45-degree angle testing, all six samples survived the cyclic loading phase. The maximum failure force in this sample was 261.96 ± 49.44 N. All failed via anchor pullout (Figure 5). When compared to the historical data of commercially available products, the 90-degree loaded samples do not show a statistical difference. The 45-degree loaded samples do show a significant difference, but were underpowered (n = 5) due to the low sample size used in the comparison paper. It is important to note that the outcomes were trending toward a marked improvement of our novel design over the commercial product. A 95% confidence interval and p-values for each comparison of the respective commercial product from historically published studies, in comparison to our results, are found in Table 2.

**Table 2:** Continuous outcome non-inferiority statistical analysis results A comparison of testing our all-suture anchor (made with BroadBand™ suture tape) to commercially available products, using published findings taken from Oh et al. and Douglass et al. [2,3]. *statistically significant PCF, Pounds per Cubic Foot; DF, Degrees of Freedom; CI, Confidence Interval

Block Angle and Load Insertion Relative to Load Direction	Block Density (PCF)	Commercial Product	Maximum Force (N)	95% CI	t-statistic (DF)	p-value
90°	20	Iconix 25	196.0 ± 15.9	-27.9 to 68.4	0.884 (17)	0.39
		Juggerknot 2.9	193.8 ± 20.2	-31.0 to 66.9	0.775 (17)	0.45
		Iconix 3	180.0 ± 16.0	-44.0 to 52.4	0.183 (17)	0.86
		Y-Knot Flex 1.8	176.1 ± 18.0	-48.3 to 48.8	0.012 (17)	0.99
	10	OmegaKnot	181.9 ± 13.7	-64.5 to 76.6	0.188 (12)	0.85
45°	10	OmegaKnot	167.8 ± 20.4	-40.4 to 148.0	-3.959 (9)	0.003*

## Discussion

Our design of the ASA has met the cost and non-inferiority aims of our study. The pullout force of the suture materials is comparable to the published historical data of commercially available ASAs. Importantly, all anchors tested in the Douglass et al. study (4/6) were deployed in a 20 PCF foam block, which is more representative of normal bone quality. Our test used a 10 PCF foam block, representative of osteoporotic bone quality, and outcomes still showed non-inferiority to the Douglass et al. outcomes [2]. We anticipate that future studies of our anchors in simulated normal bone may show even higher pullout force outcomes.

While commercially available products have improved over time and advanced the repair of soft tissue, this has come at a major expense. Depending on the institution, the cost of each ASA ranges from $200 to $2,000 for a hospital to purchase and $500 to $5,000 for patients or insurance [4]. A literature review conducted in 2023 estimated that the inflation-adjusted cost of a single surgical anchor would average $578 ± $220 [4]. The cost of our novel anchor materials is less than $25 and can be used to make multiple suture anchors. The cost of the deployment tool is less than $100 and can be sterilized for multiple uses. However, deployment tools from any commercially available suture anchor can also work for this purpose. Additionally, the materials do not require FDA approval because they are already used in patient care. Thus, we believe patients will only need disclosure of their off-label use. In regard to withstanding force, our non-inferiority analysis found that the anchors are not significantly different from commercial products in low-density bone models. This indicates our hand-made anchors are biomechanically comparable and able to withstand similar forces as other products, and may be used interchangeably.

While testing our construct, it was important to test the product under physiologic conditions, which required cyclic loading to mimic the conditions of muscle use postoperatively. Cyclic loading versus single pull-out was studied in 1997 to determine the weak link in rotator cuff repair - either the bone interface or the suture itself. In studies using a single pull-out, failure predominantly occurred via the suture anchor. When cyclic loading was used to simulate muscle attachments, failure was also seen at the bone interface, prompting the development of augmented suture anchor designs to account for the different failure types [7]. Our testing design accounted for physiologic components by using multiple angles for pullout. The 45-degree angle simulates the physiologic movement of the supraspinatus, while the 90-degree angle simulates the pull during knot tying in the operating room [3]. Additionally, by modeling our biomechanical testing after Oh et al., we were able to directly compare our ASA to the commercially available ASAs from the same study [3].

Our design also takes into consideration the testing of earlier anchor systems that similarly showed the bone and surface/tissue interface as modes of failure. The design for our anchor is structurally similar to available anchors and is simple to recreate. The advantage of these designs over rigid anchors is that they are smaller, which allows less bone to be removed with insertion and multiple insertion points along the interface for better fixation [8]. There is concern, however, that these anchors may fail due to disruption of cortical bone. With decortication of bone during joint repair or increased tunneling diameters, there may be greater rates of failure [8]. Therefore, a benefit of our design is that it requires a smaller tunneling diameter and a lower likelihood of failure due to disruption of cortical bone.

The mode of failure can be used to evaluate the anchor design. Our suture design is similar to that of the Iconix 1 and Y-knot, where the suture is woven into the anchor by a single suture, as opposed to being passed through a lumen of the anchor or having multiple sutures. In a previous study testing these anchors in shoulders with cyclic loading, their main mode of failure was via anchor pullout. This suggested that the anchor itself is less robust, as opposed to the Iconix 3, which has three strands of suture woven through a longer anchor [9]. Based on this information and the similar failure mechanism, we identified a need for further design modifications to increase the robustness of our anchor. This will also allow for better implantation into stronger bone densities, as ours was tested in osteoporotic bone density conditions. Another disadvantage of a self-made anchor during surgery is the time used to make the device - approximately two minutes - versus a pre-made, ready-to-use anchor [10]. We believe the benefit in cost reduction would outweigh the additional operating room time in most cases. These cost reductions make access more affordable for uninsured or low-income patients. A major limitation of our study is that the 45-degree angle was underpowered due to the limited sample size (n = 5) used in comparison data from the literature.

During preliminary testing, we encountered initial slippage of the suture from the Instron test grips, which required modification of the suture knot and the addition of a dowel to the test group to prevent early failure (Figure 4C). This was corrected prior to the collection of final data. Future work for our study will be completed in higher-density bone models, as well as cadaveric testing. Consideration for design adaptations in the next models may be required to increase the pullout force while not impacting the soft tissue-to-suture interface. This is strictly a biomechanical study, and it was performed in osteoporotic bone models only, not tested in cadaveric samples or patients.

## Conclusions

The biomechanical evaluation of a novel ASA demonstrated comparable performance to commercially available anchors in osteoporotic bone. Importantly, the novel anchor offers a significant cost advantage, which could make it a viable alternative in resource-limited settings. By employing testing methodologies consistent with those used in previously published studies, we facilitated direct comparisons of our findings with existing literature. Furthermore, our testing incorporated physiological conditions simulating supraspinatus and rotator cuff repairs, providing relevant biomechanical insights. It is crucial to note, however, that this study is limited to biomechanical analysis and has not yet been validated through cadaveric or clinical testing. As such, caution is warranted when considering the use of this anchor in clinical scenarios. We propose that this study serves as a prototype, laying the groundwork for more extensive research involving cadaveric models with varying bone densities to further validate its efficacy and safety.
